# Impact of a Workflow-Integrated Web Tool on Resource Utilization and Information-Seeking Behavior in an Academic Anesthesiology Department: Longitudinal Cohort Survey Study

**DOI:** 10.2196/26325

**Published:** 2021-07-26

**Authors:** Sumeet R Gopwani, Erin Adams, Alexandra Rooney, Eleni Tousimis, Katherine Ramsey, Sohan Warusha

**Affiliations:** 1 Department of Anesthesiology MedStar Georgetown University Hospital Washington, DC United States; 2 Department of Anesthesiology Weill Cornell Medical College New York, NY United States; 3 Department of Anesthesiology Brigham and Women's Hospital Boston, MA United States; 4 Department of Surgery, Breast Center MedStar Georgetown University Hospital Washington, DC United States; 5 Department of Anesthesiology Kaiser Permanente Mid-Atlantic Permanente Group Rockville, MD United States; 6 Department of Anesthesiology Temple University Hospital Philadelphia, PA United States

**Keywords:** graduate medical education, learning technology, anesthesiology, information-seeking behavior, web tool, teaching, millennial learners

## Abstract

**Background:**

Medical resident reading and information-seeking behavior is limited by time constraints as well as comfort in accessing and assessing evidence-based resources. Educational technology interventions, as the preferred method for millennial leaners, can reduce these barriers. We implemented an educational web tool, consisting of peer-reviewed articles as well as local and national protocols and policies, built into the daily workflow of a university-based anesthesiology department. We hypothesized that this web tool would increase resource utilization and overall perceptions of the educational environment.

**Objective:**

The goal of this study was to demonstrate that an educational web tool designed and built into the daily workflow of an academic anesthesia department for trainees could significantly decrease barriers to resource utilization, improve faculty-trainee teaching interactions, and improve the perceptions of the educational environment.

**Methods:**

Following Institutional Review Board approval, a longitudinal cohort survey study was conducted to assess trainee resource utilization, faculty evaluation of trainees’ resource utilization, and trainee and faculty perceptions about the educational environment. The survey study was conducted in a pre-post fashion 3 months prior to web tool implementation and 3 months following implementation. Data were deidentified and analyzed unpaired using Student *t* tests for continuous data and chi-square tests for ordinal data.

**Results:**

Survey response rates were greater than 50% in all groups: of the 43 trainees, we obtained 27 (63%) preimplementation surveys and 22 (51%) postimplementation surveys; of the 46 faculty members, we obtained 25 (54%) preimplementation surveys and 23 (50%) postimplementation surveys. Trainees showed a significant improvement in utilization of peer-reviewed articles (preimplementation mean 8.67, SD 6.45; postimplementation mean 18.27, SD 12.23; *P*=.02), national guidelines (preimplementation mean 2.3, SD 2.40; postimplementation mean 6.14, SD 5.01; *P*<.001), and local policies and protocols (preimplementation mean 2.23, SD 2.72; postimplementation mean 6.95, SD 6.09; *P*=.02). There was significant improvement in faculty-trainee educational interactions (preimplementation mean 1.67, SD 1.33; postimplementation mean 6.05, SD 8.74; *P*=.01). Faculty assessment of trainee resource utilization also demonstrated statistically significant improvements across all resource categories. Subgroups among trainees and faculty showed similar trends toward improvement.

**Conclusions:**

Learning technology interventions significantly decrease the barriers to resource utilization, particularly among millennial learners. Further investigation has been undertaken to assess how this may impact learning, knowledge retention, and patient outcomes.

## Introduction

### Barriers to Resource Utilization

Starting in 1997 with a survey of family medicine residents in internal medicine, physical medicine, and rehabilitation programs, who were followed up with in 2004, several attempts have been made to quantify and evaluate trainees’ reading behaviors [1-3]. In alignment with adult learning theory, a study conducted by Cassidy suggests that residents’ desire to learn is primarily motivated by personal interests and clinical relevance to patient care rather than by training requirements [4]. Johnson and colleagues’ investigation showed that residents read for approximately 3.7 hours per week [1]. They also showed that while trainees desired to read more, they were limited by fatigue and time constraints due to personal obligations. The groups concluded that improved delivery of educational materials, particularly via new databases and technologies, could improve the breadth and depth of resident reading. While this was true in 1997 and 2004, as the body of medical literature grows exponentially, it is even truer today.

With the extensive volume of medical literature available at the click of a mouse, one might wonder why any further learning technology intervention is needed. This expansion of electronic data, as well as fragmentation of resources across multiple websites and forums, has created a barrier to information-seeking behaviors [5,6]. A qualitative study conducted through five focus group interviews about a French general medicine program in 2015 found that both residents and general practitioners understand the importance of utilizing evidence-based medicine (EBM) and the need for unbiased information. However, study participants who generally used a limited number of online sources were not confident with their ability to assess the quality of information found, and they generally sought information in concordance with their existing knowledge [7]. Barriers to resource utilization are a problem that has been well-enough identified to have incited the development of the BARRIERS (Barriers to Research Utilization Scale) scale in 1991, which has been utilized in some 63 studies [8].

### Training Tools for Graduate Medical Education

Learning technologies in graduate medical education and anesthesiology are in their early stages but continue to grow. In a broad review of the 21st-century learner, Chandrasoma and Chu confirmed that millennial learners overwhelmingly had smartphones, so they preferred to learn via electronic methods on a variety of platforms as passive consumers as opposed to content creators [9]. Directed reading programs are one learning intervention that can be implemented as a learning technology. de Virgilio and colleagues implemented a nontechnology-based directed reading program, which, along with textbook readings, included weekly exams [3]. This resulted in increased reading and improved examination scores in a surgical residency program. A directed reading program as a learning technology, in which readings were targeted toward in-training exam objectives, has also been successfully implemented in internal medicine as well as obstetrics and gynecology residency programs, resulting in improved board pass rates [10,11].

### Use of Web Tools as a Novel Strategy for Learning

With the increased use of digital learning tools in medical education, web tools remain the most frequently utilized digital resource among medical students and residents [12]. A recent survey study of an inpatient medicine team found that web-based learning interventions improved self-directed learning, communication goals, and learning environment among medical students and residents [13].

With consideration to Thomas et al’s conceptual framework for curriculum development [14], as well as O’Brien’s conceptual framework for learning technology implementation [15], we have sought to build a learning technology into the daily workflow of trainees and faculty. These frameworks, in particular, guided us in attempting to evaluate our learners’ needs as well as the effectiveness of our learning technology in an iterative process. This learning technology consisted of an online teaching file of EBM resources as well as local and national policies and protocols that were on the same web tool as the daily operating room schedules and staff assignments. We were guided by a constructivist learning theory in our attempt to provide primary resources to trainees, which allowed them to build constructs in direct connection with their clinical experiences.

Firstly, we hypothesized that implementation of this web tool into the workflow would increase utilization of the provided resources by reducing barriers to access, including time constraints. Secondly, we hypothesized that the web tool would improve the trainees’ satisfaction with the educational environment, improve resident-faculty educational interactions, and improve faculty evaluation of trainee resource utilization.

## Methods

### Setting and Participants

Institutional Review Board approval was obtained on February 16, 2017, to conduct a longitudinal cohort survey study of trainees (ie, anesthesiology resident physicians and student registered nurse anesthetists [SRNAs]) and faculty (ie, physician anesthesiologists and certified registered nurse anesthetists [CRNAs]) at MedStar Georgetown University Hospital, a tertiary academic medical center in Washington, DC. Nonrandom sampling included the full accessible population of trainees and faculty. The primary outcome of this study was to determine if an educational web tool (Multimedia Appendix 1) for trainees designed and built into the daily workflow of an academic anesthesia department could significantly increase the trainees’ utilization of provided resources. The secondary outcome was to determine if this web tool could improve the perceptions of the educational environment, faculty evaluation of trainee recourses, and resident-faculty educational interactions.

### Survey Development

Surveys attempted to elicit information regarding the 3 months prior to, and the 3 months after, implementation of the web tool. The trainee survey (Multimedia Appendix 2) queried the number of journal articles read or referenced, the number of local and national policies referenced, trainees’ self-perceived efficiency for accessing these resources, and their overall satisfaction with their education and educational resources within the department. The faculty survey (Multimedia Appendix 3) queried the faculty members’ perception of their trainees’ use of journal articles as well as local and national policies and their perception that the department provided effective educational resources. Evaluative queries were rated on a 10-point Likert scale, ranging from 1 (strongly disagree) to 10 (strongly agree). All surveys were completed on paper and administered by two medical students working with the research team.

### Introduction of the Online Web Tool

Following the collection of the preintervention surveys, the online web tool—Departmental Intranet—was introduced, with operating room schedules and assignments published daily. Resources on the web tool were compiled and indexed by the research team. The web tool consisted of 121 journal articles, indexed in a variety of subject matter; 156 local policies and protocols; and 38 national society policies. The web tool was introduced with a brief oral presentation at the monthly faculty meeting, the resident morning lecture, and at grand rounds. A total of 3 months following the introduction of the web tool, identical postimplementation surveys were passed out to both trainee and faculty groups.

### Data Analysis

Data were compiled in a deidentified manner and analyzed as trainee and faculty composite data, as well as in subgroups. Data were analyzed unpaired using the Student *t* test for continuous data and the chi-square test for ordinal data.

## Results

### Survey Responses

Survey response rates were greater than 50% in all groups: of the 43 trainees, we obtained 27 (63%) preimplementation surveys and 22 (51%) postimplementation surveys; of the 46 faculty members, we obtained 25 (54%) preimplementation surveys and 23 (50%) postimplementation surveys (Table 1).

**Table 1:** Survey response rates for trainees and faculty in the preimplementation and postimplementation periods.

Participants		Responses preimplementation, n (%)	Responses postimplementation, n (%)
**Trainees**			
	Total (n=43)	27 (63)	22 (51)
	Residents (n=23)	15 (65)	14 (61)
	Student registered nurse anesthetists (n=20)	12 (60)	8 (40)
**Faculty**			
	Total (n=46)	25 (54)	23 (50)
	Physicians (n=27)	16 (59)	14 (52)
	Certified registered nurse anesthetists (n=19)	9 (47)	9 (47)

### Trainee Resource Utilization

**Figure 1:**
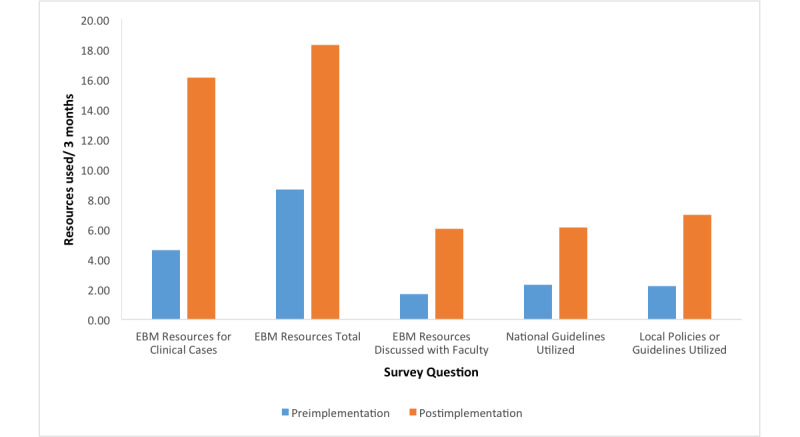
Trainee resource utilization. EBM: evidence-based medicine.

Postimplementation trainee survey results showed a significant increase in the utilization of all resource categories, including journal articles (preimplementation mean 8.67, SD 6.45; postimplementation mean 18.27, SD 12.23; *P*=.02), national guidelines (preimplementation mean 2.3, SD 2.40; postimplementation mean 6.14, SD 5.01; *P*<.001), and local policies (preimplementation mean 2.23, SD 2.72; postimplementation mean 6.95, SD 6.09; *P*=.02).  There was also significant improvement in the resources that residents referenced, specifically for their clinical cases (preimplementation mean 4.63, SD 3.75; postimplementation mean 16.09, SD 20.07; *P*=.005), as well as faculty-trainee discussions of journal articles (preimplementation mean 1.67, SD 1.33; postimplementation mean 6.05, SD 8.74; *P*=.01) (Figure 1).

**Figure 2:**
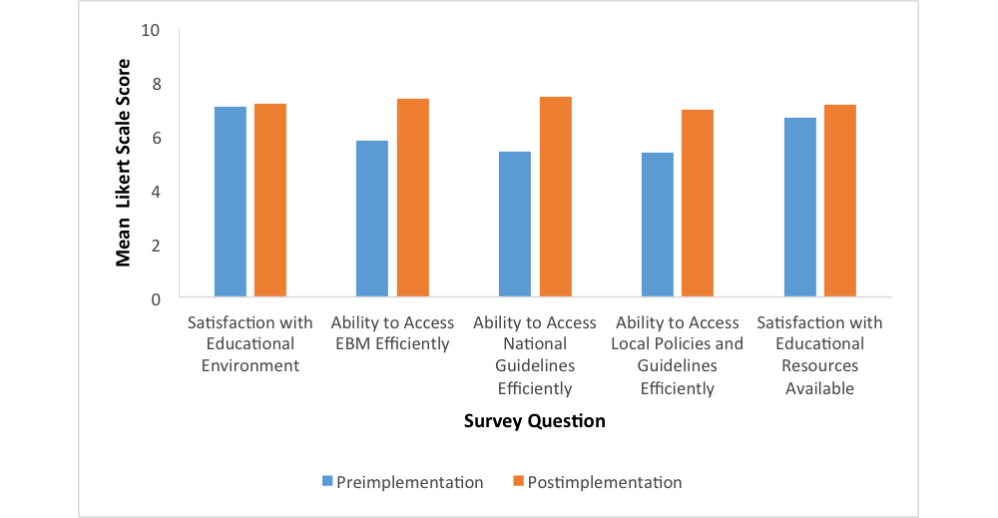
Trainee evaluation of educational environment. Questions were rated on a 10-point Likert scale, ranging from 1 (strongly disagree) to 10 (strongly agree). EBM: evidence-based medicine.

Trainees also self-reported feeling that it was more efficient to identify EBM resources in their clinical practice after implementation of the web tool (preimplementation mean 5.81; postimplementation mean 7.36; *P*=.03) (Figure 2).

### Trainee Subgroup Analysis

Subgroup analysis showed that implementation of the web tool led to broad increases in resource utilization for both SRNAs and resident physicians as well as satisfaction among both groups with the educational resources, though not all results were statistically significant (Table 2).

**Table 2:** Subgroup analysis: trainee resource utilization and trainee perceptions.

Trainee survey item	Student registered nurse anesthetists			Resident physicians		
	Pre^a^, mean (SD)	Post^b^, mean (SD)	*P* value	Pre, mean (SD)	Post, mean (SD)	*P* value
How many EBM^c^ articles have you read for your cases in the past 3 months?	4.92 (3.70)	12.63 (7.71)	.007	4.40 (3.91)	18.07 (8.64)	.04
How many EBM articles have you read in total in the past 3 months?	11.58 (6.73)	15.00 (7.60)	.30	6.33 (5.33)	20.14 (8.64)	.045
How many days have faculty discussed EBM articles in the past 3 months?	2.33 (1.30)	2.50 (1.85)	.85	1.13 (1.13)	8.07 (4.45)	.02
Satisfaction with educational environment (score^d^)	7.67 (2.02)	8.50 (1.07)	.29	6.60 (1.59)	6.43 (2.03)	.80
I can access EBM resources efficiently (score^d^)	5.92 (2.02)	8.38 (2.77)	.03	5.73 (2.79)	6.79 (2.08)	.26
How many national guidelines have you referenced in the past 3 months?	1.58 (1.88)	4.25 (3.06)	.02	2.87 (2.67)	7.21 (5.66)	.01
I can access national guidelines efficiently (score^d^)	4.58 (3.34)	7.63 (2.33)	.04	6.07 (2.37)	7.36 (1.86)	.11
How many local policies and guidelines have you referenced in the past 3 months?	3.67 (3.26)	3.00 (3.21)	.65	1.07 (1.49)	6.93 (6.96)	.003
I can access local policies and guidelines efficiently (score^d^)	5.08 (2.57)	7.38 (3.20)	.09	5.60 (2.80)	6.71 (2.89)	.31
I am satisfied with educational resources provided (score^d^)	7.00 (1.95)	8.75 (1.16)	.04	6.40 (1.40)	6.21 (2.15)	.78

^a^Pre: preimplementation.

^b^Post: postimplementation.

^c^EBM: evidence-based medicine.

^d^Survey items were rated on a 10-point Likert scale, ranging from 1 (strongly disagree) to 10 (strongly agree).

### Faculty Evaluation

**Figure 3:**
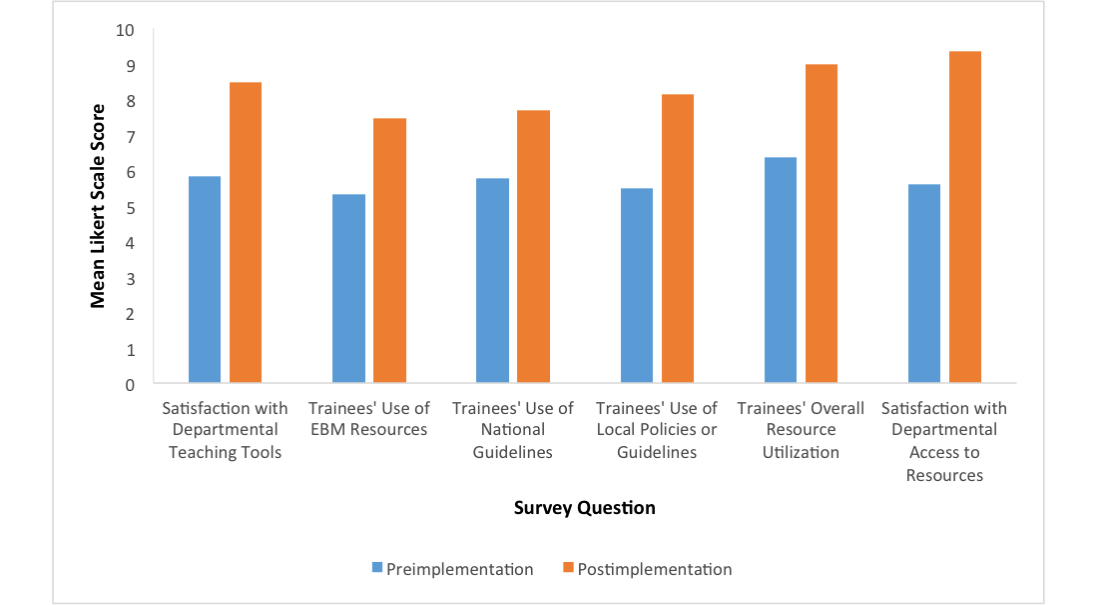
Faculty evaluation of educational environment. Questions were rated on a 10-point Likert scale, ranging from 1 (strongly disagree) to 10 (strongly agree). EBM: evidence-based medicine.

Faculty were asked to assess the trainees’ ability to efficiently utilize evidence-based articles, local policies, and national policies. Faculty reported that trainees showed an improved ability to locate resources and reported increased satisfaction with resources provided by the department (Figure 3).

### Faculty Subgroup Analysis

Subgroup analysis showed that both CRNAs and attending physicians reported similar improvements in trainee abilities to identify and utilize resources (Table 3).

**Table 3:** Subgroup analysis: faculty evaluations of trainees and faculty perceptions.

Faculty survey item	Certified registered nurse anesthetists (score^a^)			Physician anesthesiologists (score^a^)		
	Pre^b^, mean (SD)	Post^c^, mean (SD)	*P* value	Pre, mean (SD)	Post, mean (SD)	*P* value
The department provides effective teaching tools	5.78 (2.68)	8.78 (0.97)	.01	5.88 (1.93)	8.29 (1.73)	.002
Trainees effectively locate and implement EBM^d^ resources	4.89 (2.71)	7.33 (1.12)	.02	5.56 (2.39)	7.57 (2.10)	.02
Trainees effectively locate and implement national guidelines	6.00 (2.40)	7.11 (1.62)	.26	5.63 (2.53)	8.07 (1.73)	.005
Trainees effectively locate and implement local policies and guidelines	5.33 (2.06)	8.33 (1.41)	.002	5.56 (2.48)	8.00 (1.57)	.003
Trainees can be effectively directed to the above resources	6.56 (1.59)	8.67 (1.32)	.007	6.25 (2.11)	9.21 (1.05)	<.001
The department has an effective system for faculty to access the above resources	5.22 (3.23)	9.44 (0.88)	.002	5.81 (2.79)	9.29 (1.14)	<.001

^a^Survey items were rated on a 10-point Likert scale, ranging from 1 (strongly disagree) to 10 (strongly agree).

^b^Pre: preimplementation.

^c^Post: postimplementation.

^d^EBM: evidence-based medicine.

## Discussion

### Principal Findings

Following the implementation of the web tool, our trainees showed a significant improvement in the utilization of peer-reviewed articles, national guidelines, and local policies and protocols. Postimplementation surveys demonstrated a significant improvement in faculty-trainee educational interactions as well as faculty assessment of trainee resource utilization.

The development of an online web tool, more specifically, a centralized repository of academic articles, local policies and protocols, and national policies, was considered a first step in the implementation of improved educational technology at our institution. While there is certainly an abundance of materials available to faculty and trainees alike, searching and indexing through an ever-growing volume of information can be daunting. The goal of our web tool was to build these resources into the daily workflow, decrease barriers to utilization, and create a base for further educational technology interventions.

### Adult Learning Theory for Millennials

The implementation of a departmental educational web tool is an example of applying adult learning theory for the millennial learner. Adult learning theory tells us that our residents are not oriented toward classical methods of lecture-based education with a postponed application of knowledge. Alternatively, they are oriented toward educational materials that are integrated into their current clinical experience and the immediacy of application. For this reason, modern e-learning in a professional environment is asynchronous, personalized, and just-in-time [16]. Millennial learners in the digital age prefer modalities that are online and self-paced. Meeting these needs requires us to build relevant educational tools into the learning environment [17]. Our survey study shows that creating such tools is an effective way to connect with millennial adult learners via their preferred methods. By meeting them where they are, we are able to encourage our learners to read, to improve their satisfaction with the educational environment, and to better connect trainees and faculty.

### Integration Into the Workflow

We focus on the concept of integrating our web tool into the daily workflow of our learners because this method approximates a passive clinical decision support (CDS) system. By providing resources to providers based on the patients they are seeing, CDS systems ease the barriers to information-seeking behaviors, as discussed earlier [18]. CDS systems have been used in our field of anesthesiology to encourage protocol utilization, remind providers to administer antibiotics, and to improve compliance and billing [19-21]. Active CDS systems send notifications to providers, while passive systems like ours require providers to click links to access information [22]. Our web tool is not a full CDS system. The web tool lacks the data acquisition and rules modules to be considered an independent CDS system. However, by taking the relevant resources, including local and national protocols, and linking them directly into the workflow, we lessen the barriers to utilization of these resources and approximate a CDS system with many of the benefits. CDS systems have been shown to be very effective in increasing protocol utilization, with findings similar to our web tool implementation [21,22].

### Limitations

The primary use of self-reported resource utilization via surveys introduces certain limitations and the potential for bias. Surveys were administered in person, preventing false respondents. Surveys were administered by medical students and were deidentified to minimize *social desirability* bias; however, this cannot be eliminated and may be particularly prevalent among trainees. Given that survey response rates were 50%, there was a possibility of self-selection bias in which trainees and faculty that did not find the intervention helpful may have elected not to respond, which would skew results in the positive direction. Additionally, recall bias must be considered when asking respondents about their past behaviors, even over a 3-month period.

Utilizing data such as web tool click rates or log-in data would have provided more “hard” data regarding resource utilization. Survey response rates were acceptable at 50% but could have been higher; in addition, due to the variance in pre- and postsurvey respondents, and the desire to deidentify data, the analysis was performed using an unpaired method. Additionally, this survey study looked at only one pre- and postimplementation time point and, therefore, says nothing about the long-term use of resources, and certainly says nothing about an improvement in knowledge gain or educational achievement. An underlying assumption is that more resource utilization is better, though this may be unproven.

### Future Studies

As we continue to build electronic resources to assist in anesthesiology training, further investigations should ascertain what the ideal volume and type of resources would be for such a web tool, and what delivery mechanisms are ideal to introduce these resources to trainees and encourage utilization. Additionally, determinations can be made for what sorts of systems will decrease barriers to faculty-trainee teaching. This group has begun to undergo further investigations to deliver resources from this web tool to providers in a more targeted method utilizing a CDS system. We have begun evaluation of how such targeted delivery methods increase utilization of policies, improve resident reading, and improve trainee knowledge acquisition and retention.

### Conclusions

As we hypothesized, the implementation of our web tool did increase the volume of journal articles, local policies, and national policies that our trainees utilized. The web tool also subjectively increased the self-rated comfort of trainees in their utilization of resources, as well as the faculty evaluation of trainee resource utilization. Encouragingly, this common framework for access to shared resources also increased the volume of faculty-trainee discussions about evidence-based resources. We are encouraged with the belief that building from this, such a framework can ease barriers to faculty-trainee educational interactions.

## Appendix

Multimedia Appendix 1 Sample screenshots of the web tool.

Multimedia Appendix 2 Trainee survey.

Multimedia Appendix 3 Faculty survey.

## Abbreviations

BARRIERS: Barriers to Research Utilization Scale
CDS: clinical decision support
CRNA: certified registered nurse anesthetist
EBM: evidence-based medicine
SRNA: student registered nurse anesthetist

## References

[1] Johnson KH, Dayrit M, Bazargan M (1997). The reading habits of family practice residents. Fam Med.

[2] Lai CJ, Aagaard E, Brandenburg S, Nadkarni M, Wei HG, Baron R (2006). Brief report: Multiprogram evaluation of reading habits of primary care internal medicine residents on ambulatory rotations. J Gen Intern Med.

[3] de Virgilio C, Stabile BE, Lewis RJ, Brayack C (2003). Significantly improved American Board of Surgery In-Training Examination scores associated with weekly assigned reading and preparatory examinations. Arch Surg.

[4] Cassidy K (2004). The adult learner rediscovered: Psychiatry residents' push for cognitive-behavioral therapy training and a learner-driven model of educational change. Acad Psychiatry.

[5] Crotty BH, Mostaghimi A, Reynolds EE (2012). Adoption of a wiki within a large internal medicine residency program: A 3-year experience. J Am Med Inform Assoc.

[6] Currie LM, Graham M, Allen M, Bakken S, Patel V, Cimino JJ (2003). Clinical information needs in context: An observational study of clinicians while using a clinical information system. AMIA Annu Symp Proc.

[7] Schuers M, Griffon N, Kerdelhue G, Foubert Q, Mercier A, Darmoni SJ (2016). Behavior and attitudes of residents and general practitioners in searching for health information: From intention to practice. Int J Med Inform.

[8] Funk SG, Champagne MT, Wiese RA, Tornquist EM (1991). BARRIERS: The barriers to research utilization scale. Appl Nurs Res.

[9] Chandrasoma J, Chu LF (2016). Teaching the 21st century learner: Innovative strategies and practical implementation. Int Anesthesiol Clin.

[10] Drake SM, Qureshi W, Morse W, Baker-Genaw K (2015). A time-efficient web-based teaching tool to improve medical knowledge and decrease ABIM failure rate in select residents. Med Educ Online.

[11] Chern I, Antalan C, Aeby T, Hiraoka M (2018). The effect of a targeted educational activity on obstetrics and gynecology resident in-training examination scores. Hawaii J Med Public Health.

[12] Torous J, O'Connor R, Franzen J, Snow C, Boland R, Kitts R (2015). Creating a pilot educational psychiatry website: Opportunities, barriers, and next steps. JMIR Med Educ.

[13] Carson TY, Hatzigeorgiou C, Wyatt TR, Egan S, Beidas SO (2020). Development and implementation of a web-based learning environment for an inpatient internal medicine team: Questionnaire study. JMIR Med Educ.

[14] Thomas PA, Kern DE, Hughes MT, Chen BY (2015). Curriculum Development for Medical Education: A Six-Step Approach. 3rd edition.

[15] O'Brien JA (1994). Introduction to Information Systems. 7th edition.

[16] Novak GM, Patterson ET, Gavrin AD, Christian W (1999). Just-In-Time Teaching: Blending Active Learning with Web Technology.

[17] Wolpaw JT, Uhlig E, Isaac GR, Dwivedi P, Lekowski RW, Toy S (2020). Anesthesia learning in the digital age: Are program directors and residents on the same page?. J Educ Perioper Med.

[18] Epstein RH, Dexter F, Patel N (2015). Influencing anesthesia provider behavior using anesthesia information management system data for near real-time alerts and post hoc reports. Anesth Analg.

[19] McDonald CJ (1976). Protocol-based computer reminders, the quality of care and the non-perfectability of man. N Engl J Med.

[20] Nair BG, Newman S, Peterson GN, Schwid HA (2011). Automated electronic reminders to improve redosing of antibiotics during surgical cases: Comparison of two approaches. Surg Infect (Larchmt).

[21] Kooij FO, Klok T, Hollmann MW, Kal JE (2008). Decision support increases guideline adherence for prescribing postoperative nausea and vomiting prophylaxis. Anesth Analg.

[22] Nair BG, Gabel E, Hofer I, Schwid HA, Cannesson M (2017). Intraoperative clinical decision support for anesthesia: A narrative review of available systems. Anesth Analg.

