# Supplementary material for: Impact of a Workflow-Integrated Web Tool on Resource Utilization and Information-Seeking Behavior in an Academic Anesthesiology Department: Longitudinal Cohort Survey Study
Source: JMIR Med Educ. 2021 Jul 26;7(3):e26325. doi: 10.2196/26325 (PMC8367122; doi:10.2196/26325)
Supplement: Multimedia Appendix 1 [file mededu_v7i3e26325_app1.pdf]

TEACHING FILE POLICIES & PROCEDURES

MedStar Health

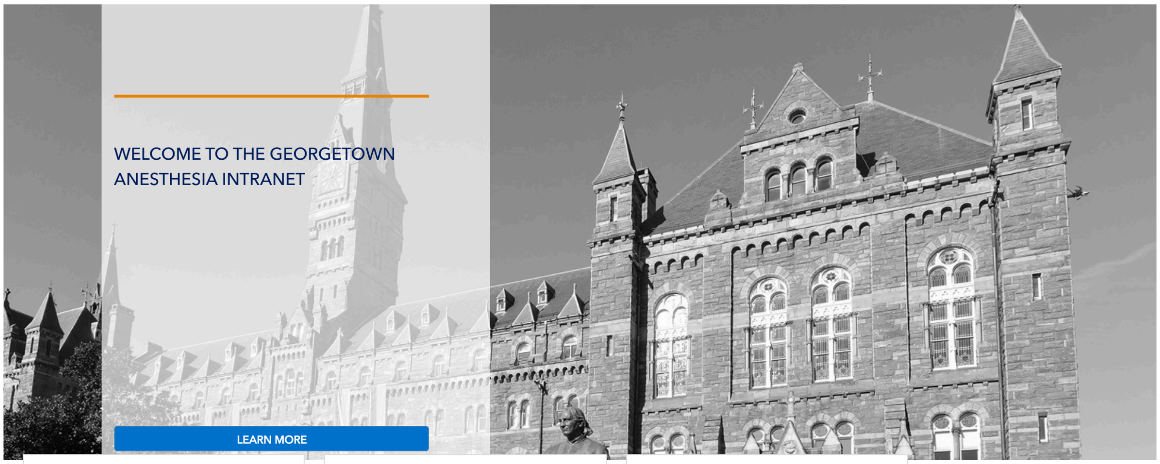

Contact List

Anesthesia Assignments

Click below to see Anesthesia Staff Assignments

Anesthesia Schedule

Click below to see Anesthesia Schedule

TEACHING FILE POLICIES & PROCEDURES

MedStar Health

Home > Policies and Procedures

Share this page: A A A

Policies and Procedures

MedConnect and Job Aids

Resolving MedConnect/MRDI Deficiency

How to Add Acute Pain Service Autotext

Autotext in Pain Service Notes

MedConnect Powerpoint

National Policies

ACLS/PALS

ACLS Cardiac Arrest/VFib/VTach

ACLS Tachycardia With a Pulse

ACLS Immediate Post-Arrest

Pediatric BLS

PALS Tachycardia

PALS Respiratory Emergencies

ACLS Bradycardia

ACLS Acute Coronary Syndrome

ACLS Stroke

PALS Cardiac Arrest

PALS Bradycardia

Anticoagulation Guidelines

ASRA 3rd Ed. Anticoagulation Guidelines

ASRA 4th Ed. Update - Antiplatelet Agents

ASRA LA Toxicity Checklist

ASRA 3rd Ed/Horlocker Neuraxial Summary Recommendations

NYSORA Anticoagulation Guidelines

ASA Practice Guidelines

Anesthetic Monitoring Standards

Ethical Guidelines for Anesthetic Care

Management of Respiratory Depression

Management of Obstructive Sleep Apnea

Perioperative Blood Management

Perioperative TEE

Acute Pain Management Guidelines

ASA Physical Status Classification

Preoperative Fasting Guidelines

Prevention and Management of OR Fires

Practice Guidelines for Difficult Airway

Post anesthetic Care Guidelines

Neuraxial Anesthesia in Obstetrics

Chronic Pain Management Guidelines

What's New!

ePreOp Job Aid

Pre-op Management of Chronic Medications

Perioperative Anticoagulation Guidelines

General Anesthesia

- Perioperative Pulmonary Dysfunction
- Perioperative AKI
- Perioperative Glycemic Control
- Perioperative Steroid Replacement
- Airway Trauma
- Malignant Hyperthermia
- Perioperative Stroke
- Perioperative Nutrition
- Temperature Management
- Perioperative Visual Loss
- Perioperative Beta-Blockade
- Smoking Cessation
- Geriatric Anesthesia
- Perioperative Ventilation Strategies
- Regional Anesthesia in Opthalmologic Surgery
- B-AWARE Trial (Intraoperative Awareness)
- B-UNAWARE Trial (Intraoperative Awareness)

Pharmacology

- Opioid Pharmacology
- Inotropes and Vasopressors
- Vasopressin
- Etomidate
- Anaphylaxis
- Cephalosporins in Penicillin Allergy
- Methylene Blue
- Propofol Pain
- Peri-anesthetic Anaphylaxis

Monitoring and Anesthesia Equipment

- Blood Pressure Monitoring
- Breathing Circuits
- Tissue Oximitry
- Bispectral Index Monitoring
- Pulmonary Artery Catheters

Critical Care Medicine

- Ventilation Strategies
- Weaning from Mechanical Ventilation
- Non-invasive Mechanical Ventilation
- Renal Replacement Therapy
- ICU Sedation
- Early Goal Directed Therapy

Anesthesia and Co-Existing Disease

- Management of OSA
- Perioperative Pheochromocytoma
- Parkinsons Disease and Anesthesia
- Multiple Sclerosis
- Myasthenia Gravis
- Thymectomy in Myasthenia
- Anesthesia and HIV
- Endocrine Surgery
- Dysautonomia

Obstetric Anesthesia

- Labour Analgesia Outcomes
- Non-Obstetric Surgery During Pregnancy
- Patient Controlled Epidural Analgesia
- Post Dural Puncture Headache

Teaching File

Cardiac Disease in Anesthesia

Cardiac Complications in Non-Cardiac Surgery
Cardiac Risk in Non-Cardiac Surgery
Adverse Events with Coronary Stent
Coronary Stent Management
Hypertrophic Cardiomyopathy
Peri-Operative Management of Heart Failure
CARP Trial - Coronary Revascularizton
Pulmonary Hypertension

Cardiothoracic Anesthesia

Cardiopulmonary Bypass
Coronary Artery Bypass Grafting
Valvular Disease
Blood Pressure Management During CEA
Thoracic Aneurysm
CSF Drainage for TAA
Carotid Stenting vs Endarterectomy
Anesthesia in Thoracic Surgery
Acute Lung Injury in Thoracic Surgery
Anaesthetic Agents for Thoracic Surgery
Fluid Management in Thoracic Surgery
Preoxygenation
Pro: Low Tidal Volume During OLV
Con: Low Tidal Volumes During OLV
Ventilation Strategies for OLV
Hypoxemia During OLV
Pulmonary Dysfunction and Protection
Fiberoptic Positioning of Double Lumen Tubes
Positioning of Double Lumen Tubes
Thoracic Epidural Anaesthesia

Fluid Management

Fluid Management in ERAS
Fluid Therapy in Sepsis
Resucitation Fluids
Crystalloids vs Colloids
Hydroxyethyl Starches
SAFE Trial (albumin)

General Anesthesia

Perioperative Pulmonary Dysfunction
Perioperative AKI
Perioperative Glycemic Control
Perioperative Steroid Replacement

What's New!

ePreOp Job Aid

Pre-op Management of Chronic Medications
Perioperative Anticoagulation Guidelines

Obstetric Anesthesia

Labour Analgesia Outcomes

Non-Obstetric Surgery During Pregnancy

Patient Controlled Epidural Analgesia

Post Dural Puncture Headache

Amniotic Fluid Embolism

Epidural Hematoma

2015 Gerard W Ostheimer Whats New in OB Anesthesia

Pediatric Anesthesia

Pediatric URI

Down Syndrome

Apnea in Former Preterm Infants

Pyloric Stenosis

Congenital Cardiac Disease

Pediatric Sedation

Peri-Operative Care

Pacemakers and AICD Review

Pacemaker/AICD Algorithm

Magnet Instructions

Biotronik Pacemaker

Biotronik AICD

Boston Scientific Electrocautery Education

Boston Scientific Pacemaker

Boston Scientific AICD

Boston Scientific S-ICD

Medtronic EMI Education

Medtronic Magnet Application

St. Jude Pacemaker

St. Jude AICD

Transfusion Medicine

Transfusion vs Anemia

Blood Management

Transfusion Risks

Infectious Transfusion Risks

Transfusion Related Acute Lung Injury

Coagulopathy in Massive Transfusion

Preoperative Acute Normovolemic Hemodilution

Perioperative Cell Salvage

TRIC Trial

Post-Operative Management

Delayed Emergence

Post-operative Nausea and Vomiting

Post-operative Urinary Retention

Post-operative Cognitive Dysfunction

Post-operative Delirium

Post-operative Neurologic Complications

Music and Post-operative Recovery

Ethics

Informed Consent

IRB and Research Ethics

Peri-operative DNR

Perioperative Jehovahs Witness

Physician Wellness

Substance Abuse in Anesthesia

Fatigue in Anesthesia
