# Supplementary material for: Impact of a Workflow-Integrated Web Tool on Resource Utilization and Information-Seeking Behavior in an Academic Anesthesiology Department: Longitudinal Cohort Survey Study
Source: JMIR Med Educ. 2021 Jul 26;7(3):e26325. doi: 10.2196/26325 (PMC8367122; doi:10.2196/26325)
Supplement: Multimedia Appendix 2 [file mededu_v7i3e26325_app2.pdf]

GT Intranet Trainee Survey

Below are several questions. Please answer them to the best of your memory based on the last **3 months.**

Name: ________________________

1. What have been your clinical rotations for the past 3 months (including site)?

____

2. How many journal articles have you read or referenced, *in relation to your clinical cases*, in the past three months?

____

3. How many journal articles have you read or referenced, *in total*, in the past three months?

____

4. How many days have you discussed journal articles relevant to your clinical cases with anesthesia faculty in the past three months?

____

5. How satisfied are you with the overall clinical teaching you have received from faculty in the past 3 months?

Unsatisfied---Very Satisfied
1 2 3 4 5 6 7 8 9 10

5. You feel that it is time efficient to look up **journal articles** related to your cases.

Strongly Disagree--Strongly Agree
1 2 3 4 5 6 7 8 9 10

6. How many times in the past three months have you referenced a national guideline?

____

7. I know how to find national guidelines, and can access them efficiently.
Strongly Disagree--Strongly Agree
1 2 3 4 5 6 7 8 9 10

8. How many times in the past three months have you referenced a local policy/guideline?

____

9. I know how to find local policies/guidelines, and can access them efficiently.
Strongly Disagree--Strongly Agree
1 2 3 4 5 6 7 8 9 10

10. You feel that your patient care would improve if you had the ability to review more journal articles and policies, or better access to resources.

Strongly Disagree--Strongly Agree

1 2 3 4 5 6 7 8 9 10

11. The department provides adequate resources to assist in trainee education.

Strongly Disagree--Strongly Agree

1 2 3 4 5 6 7 8 9 10

12. If you have participated in breast surgery in the past 3 months, are you familiar with the Breast ERAS protocol?

Yes No N/A

13. What resources do you typically use to find **journal articles** related to your cases? (Such as Google, Pubmed, etc)

____
