# Supplementary material for: Impact of a Workflow-Integrated Web Tool on Resource Utilization and Information-Seeking Behavior in an Academic Anesthesiology Department: Longitudinal Cohort Survey Study
Source: JMIR Med Educ. 2021 Jul 26;7(3):e26325. doi: 10.2196/26325 (PMC8367122; doi:10.2196/26325)
Supplement: Multimedia Appendix 3 [file mededu_v7i3e26325_app3.pdf]

GT Intranet Faculty Survey

Below are several questions. Please answer them to the best of your memory based on the last **3 months.**

Name: ______________________________

1. How many journal articles have you asked trainees to read or reference in the past three months?

____

2. You feel that the department has provided effective tools, to assist you in providing trainee education.

Strongly Disagree--Strongly Agree

1 2 3 4 5 6 7 8 9 10

3. The trainees I work with effectively locate *journal articles* and evidence based resources, and apply them clinically.

Strongly Disagree--Strongly Agree

1 2 3 4 5 6 7 8 9 10

4. The trainees I work with effectively locate *national guidelines*, and apply them as needed.

Strongly Disagree--Strongly Agree

1 2 3 4 5 6 7 8 9 10

5. The trainees I work with effectively locate *local policies/guidelines*, and apply them.

Strongly Disagree--Strongly Agree

1 2 3 4 5 6 7 8 9 10

6. I am able to direct trainees to the above resources, and they are able to access them reliably.

Strongly Disagree--Strongly Agree

1 2 3 4 5 6 7 8 9 10

7. The department has an effective system to allow me to access the above resources.

Strongly Disagree--Strongly Agree

1 2 3 4 5 6 7 8 9 10
